# Radiofrequency ablation is beneficial in simultaneous treatment of synchronous liver metastases and primary colorectal cancer

**DOI:** 10.1371/journal.pone.0193385

**Published:** 2018-03-15

**Authors:** Joost Hof, Hanneke J. Joosten, Klaas Havenga, Koert P. de Jong

**Affiliations:** 1 Department of Hepato-pancreato-biliary surgery and Liver transplantation, University Medical Center Groningen, University of Groningen, Groningen, The Netherlands; 2 Department of Surgery, University Medical Center Groningen, University of Groningen, Groningen, The Netherlands; University Hospital Oldenburg, GERMANY

## Abstract

**Background:**

In patients with resectable synchronous colorectal liver metastases (CRLM), either two-staged or simultaneous resections of the primary tumor and liver metastases are performed. Data on radiofrequency ablation (RFA) for the treatment of CRLM during a simultaneous procedure is lacking. The primary aim was to analyze short-term and long-term outcome of RFA in simultaneous treatment. A secondary aim was to compare simultaneous resection with the colorectal-first approach.

**Methods:**

Retrospective analysis of 241 patients with colorectal cancer and synchronous CRLM between 2000–2016. Median follow-up was 36.1 months (IQR 18.2–58.8 months). A multivariable analysis was performed to analyze the postoperative morbidity, using the comprehensive complication index. A propensity matched analysis was performed to compare survival rates.

**Results:**

In multivariable analysis, the best predictor of lower complication severity was treatment with RFA (p = 0.040). Higher complication rates were encountered in patients who underwent an abdominoperineal resection (p = 0.027) or age > 60 years (p = 0.022). The matched analysis showed comparable overall survival in RFA treated patients versus patients undergoing a liver resection with a five year overall survival of 39.4% and 37.5%, respectively (p = 0.782). In a second matched analysis, 5-year overall survival rates in simultaneously treated patients (43.8%) was comparable to patients undergoing the colorectal first approach (43.0%, p = 0.223).

**Conclusions:**

RFA treatment of CRLM in simultaneous procedures is associated with a lower complication severity and non-inferior oncological outcome as compared to partial liver resection. RFA should be considered a useful alternative to liver resection.

## Introduction

About 20% of patients with colorectal cancer (CRC) already have liver metastases at the time of presentation of their primary tumor. The most widely used intentionally curative approach for the treatment of both tumor locations is a staged procedure with resection of the primary tumor first, followed by liver surgery at a later stage [1]. As alternative, the liver-first staged procedure was introduced in 2008 and was proposed to prevent progression of the liver metastases during the interval between resection of the primary and the liver metastases [2,3]. The third treatment option is to perform simultaneous surgery, in which the primary colorectal cancer and the liver metastases are resected during the same operative session.

Simultaneous resection of the primary CRC and synchronous CRLM has been shown to be feasible with an acceptable complication rate compared to the colorectal-first approach [4–7]. These studies score complications based on the Clavien-Dindo classification, mainly comparing the incidence of major complications [8]. However, selection of only patients in a good clinical condition contributes to this outcome. Because comorbidity and impact of surgery are predictors for developing complications, the decision to perform two major procedures simultaneously or a staged procedure is important [9–11]. As an alternative for liver resection, radiofrequency ablation (RFA) of CRLM has been shown to be associated with lower morbidity and mortality and comparable survival rates [12–15].

Strikingly, in a recent multidisciplinary international consensus paper about treatment strategy for synchronous liver metastases, RFA as a treatment option of CRLM was not even mentioned [16]. This is due to lack of evidence, and studies in this field are certainly warranted. Therefore, the aim of the present study is to analyze patients undergoing simultaneous treatment of primary CRC and CRLM, focusing on the role of RFA in short-term complications. Furthermore, a matched analysis is performed to study survival rates in RFA-treated patients vs. patients who underwent a liver resection. The last aim is to analyze survival rates in matched patients who underwent a simultaneous procedure vs. colorectal-first procedure.

## Patients and methods

The Department of Surgery of the University Medical Centre Groningen (UMCG) is a secondary and tertiary referral centre for patients with advanced colorectal cancer including CRLM in the North-Eastern part of the Netherlands. An analysis was performed using a prospectively maintained database of all patients with CRLM, in which for this study a selection of patients with synchronous liver metastases was used. The procedures, performed between January 2000 and September 2016, consisted of surgery aiming at radical resection of the primary CRC and radical resection and/or ablation of liver metastases. Only surgical resections in patients with microscopic tumor-free margins (R0) were included in the analysis. Each patient was discussed in a tumor board of hepato-pancreato-biliary and colorectal surgeons, gastroenterologists, radiologists, radiation oncologists, pathologists and medical oncologists. Most patients who underwent the colorectal-first procedure are treated for colorectal cancer in a primary hospital. Another reason for not performing simultaneous surgery is comorbidity or large liver resections (>70% of liver volume). In simultaneous procedures, we always performed the liver procedure first and the colorectal surgery second. The total duration of the procedure included anesthesia induction time. A proportion of patients with rectal cancer underwent neoadjuvant chemoradiotherapy, including 5x5 Gy radiotherapy and 6 cycles of capecitabine/oxaliplatin/bevacizumab [17,18]. Patients with more advanced liver metastases, in whom radical liver surgery was questionable, received neoadjuvant chemotherapy. They were evaluated after two or three cycles of chemotherapy, and no further cycles were administered if the computer tomography (CT) scan showed that a R0 resection was possible. Two or three additional cycles were given in patients with insufficient response.

During all simultaneous procedures, intraoperative RFA was performed under ultrasound guidance, using the RF 3000 TM Radio Frequency Ablation System (Boston Scientific, Marlborough, MA, USA) according to the manufacturers’ instructions. Depending on the size of the CRLM, the 2.0, 3.5, 4.0 or 5.0 cm diameter LeVeen electrodes were inserted. Percutaneous RFA, which was only applied in the staged approaches, was performed CT guided. In general, RFA was contraindicated in CRLMs with a diameter > 5 cm. Ablation site recurrences were defined as described earlier [19].

The number of CRLM and the size of the largest CRLM are based on pathological findings of resection specimens and on CT scans in case of RFA treatment. Follow-up consisted of a 3–4 monthly survey in the first 2 years after surgery, and 6 monthly thereafter. Follow-up consisted of serum carcinoembryonic antigen-level (CEA), liver ultrasound and thoracic X-ray, or a multiphase contrast enhanced CT-scan or MRI scan. If equivocal results of CT/MRI scan were obtained, positron emission tomography with [F-18]-fluorodeoxyglucose CT (FDG-PET-CT) was performed.

Postoperative complications were scored in the first 90 days after surgery and were categorized into general complications (for instance urinary tract or pulmonary complications), bowel-related complications (anastomotic leakage, hematoma or abscess) and liver-related complications (biloma, liver abscess). Since more than one complication can occur in the postoperative period, we applied the comprehensive complication index (CCI) [20]. This index integrates all complications and, on top of that, includes a grading of severity of all complications. This is especially relevant because apart from general complications (not related to the surgical procedure itself), both the liver procedure and the colorectal procedure can have its associated complications with variable severity.

### Ethics statement

This study was approved by the local Medical Ethical committee (METc2015/343), and was judged not to be within the scope of the Medical Research in Human Subjects Act (WMO).

In our retrospective study concerning oncological disease, it is impossible to obtain written consent of all patients, since about 50% of patients already died of the disease at the time of writing the manuscript.

We are, as medical doctors, bound by the law of confidentiality to keep all patient information fully secret. This implies all authors, as researchers, are subject to this law. The local ethics committee approved our method of including patients in our observational, retrospective study, provided that we obey the Dutch law regulations. Because we cannot obtain patient consent because of the aforementioned reasons, we cannot share the data because of patient confidentiality.

The ethics committee that approved our study:

Medical Ethical Review Board, PO Box 30.001, 9700 RB Groningen, The Netherlands, E-mail: metc@umcg.nl.

### Statistical analysis

Summary statistics are presented as percentages, median (interquartile range, IQR) or mean (± standard deviation, SD). Non-parametrical tests (chi-square test, Mann-Whitney test and Kruskall-Wallis H and paired equivalents) were applied when appropriate. Regression analyses were performed to determine the risk factors for developing any complication (complication rate) and for developing a higher comprehensive complication index (CCI) [20]. Factors with a p-value < 0.17 in univariable analysis were entered into the multivariable model. Hazard ratios and 95% CI are reported.

Survival rates were estimated using the Kaplan-Meier method with the stratified log-rank test for matched comparisons. In order to compare survival, a propensity score matching was used to reduce the influence of selection bias. A binary logistic regression was performed to predict the probability of belonging to the RFA or non-RFA treatment group, and colorectal first vs. simultaneous group. Covariates used for matching were location of the primary tumor, type of colorectal surgery, major/minor liver surgery, type of liver procedure, sex, age, neoadjuvant chemotherapy and clinical risk score [21]. We used nearest-neighbour matching, using a 1:1 ratio, with a caliper fixed to 0.2.

In all analyses, a p-value < 0.05 was considered significant. Statistical analyses were performed with IBM SPSS Statistics V22 (IBM, Armonk, New York, USA) and R software [22] using the MatchIt package [23].

## Results

### Demographics

In the study period January 2000—September 2016, a total 574 patients with colorectal liver metastases underwent 904 liver procedures, which consisted of resection, RFA or a combination of both. In the same period, 241 patients presented with synchronous liver metastases which were treated surgically. Median follow-up of all 241 patients with synchronous liver metastases was 36.1 months (IQR 18.2–58.8 months).

Patients who underwent a liver-first approach were excluded from further analyses due to low number of patients (n = 15). In the remaining 226 patients, 106 underwent the simultaneous approach and 120 underwent the colorectal-first approach. First we analyzed the 106 patients receiving the simultaneous approach. Table 1 shows the clinicopathological characteristics of all 106 patients in the simultaneous group. Neoadjuvant chemotherapy consisted in 88.4% (61 out of 69 patients) of the baseline treatment of capecitabine and oxaliplatin, of which 51 patients also received bevacizumab. Major liver resections (≥ 3 liver segments) were performed in 3 of the 24 patients who underwent an APR versus 25 of the 82 patients who underwent non-APR procedures (p = 0.079).

**Table 1:** Clinicopathological characteristics of the patients undergoing simultaneous treatment of the primary colorectal carcinoma and liver metastases.

	Total	Liver resection	Liver resection + RFA	RFA alone	P value
**Number of patients**	106	59	34	13	
**Patient characteristics**					
Mean age ± SD	61.3 ± 11.5	63.0 ± 11.3	58.8 ± 11.0	59.8 ± 13.4	0.210
Male gender	59 (55.7%)	33 (55.9%)	19 (55.9%)	7 (53.8%)	0.990
**Comorbidities**					
BMI > 30	14 (13.9%)	6 (10.3%)	7 (21.9%)	1 (9.1%)	0.282
Smoking	22 (21.2%)	14 (24.1%)	5 (15.2%)	3 (23.1%)	0.591
ASA score ≥ 3	15 (15.0%)	10 (17.5%)	5 (15.6%)	0 (0%)	0.326
Cardiovascular medication	47 (44.3%)	25 (42.4%)	15 (44.1%)	7 (53.8%)	0.752
Diabetic medication	5 (4.7%)	1 (1.7%)	2 (5.9%)	2 (15.4%)	0.101
Syst. corticosteroid medication	6 (5.7%)	1 (1.7%)	5 (14.7%)	0 (0%)	0.021
Obstructive lung disease	10 (9.4%)	6 (10.2%)	3 (8.8%)	1 (8.3%)	0.952
**Tumor characteristics**					
Rectal primary	71 (67.0%)	36 (61.0%)	24 (70.6%)	11 (84.6%)	0.226
N+ disease	66 (62.3%)	40 (67.8%)	21 (61.8%)	5 (38.5%)	0.142
Diameter CRLM in cm (median, IQR)	2.2 (1.5–3.5)	2.5 (1.5–4.0)	2.0 (1.2–3.3)	1.7 (0.9–2.7)	0.092
>1 CRLM	74 (69.8%)	34 (57.6%)	34 (100%)	6 (46.2%)	<0.001
Bilobar disease	35 (33.0%)	7 (11.9%)	26 (76.5%)	2 (15.4%)	<0.001
**Preoperative factors**					
Neoadjuvant chemotherapy	69 (66.0%)	34 (57.6%)	28 (82.4%)	7 (53.8%)	0.036
Low clinical risk score (0–2) [21]	57 (53.8%)	33 (55.9%)	15 (44.1%)	9 (69.2%)	0.268
**Surgery**					
Surgery > 8 hours	58 (55.2%)	32 (54.2%)	20 (58.8%)	6 (50.0%)	0.846
Blood loss > 500ml	49 (48.5%)	25 (45.5%)	17 (50.0%)	5 (41.7%)	0.705
**Extent of liver surgery**					<0.001
≥ 3 segments	28 (26.4%)	23 (39.0%)	5 (14.7%)	-	
1 or 2 segments	18 (17.0%)	12 (20.3%)	6 (17.6%)	-	
Local treatment	60 (56.6%)	24 (40.7%)	23 (67.6%)	13 (100%)	
• Wedge resection	24	24	-	-	
• RFA	13	-	-	13	
• RFA + wedge resection	23	-	23	-	
**Type of colorectal surgery**					0.460
APR	24 (22.6%)	12 (20.3%)	7 (20.6%)	5 (38.5%)	
LAR	48 (44.3%)	25 (42.4%)	17 (50.0%)	6 (38.5%)	
Colon	34 (33.0%)	22 (37.3%)	10 (29.4%)	2 (23.1%)	

RFA = radiofrequency ablation, APR = abdominoperineal resection, LAR = low anterior resection, N+ = lymph node positive primary tumor, ASA-score = American Society of Anaesthesiologists physical status classification system.

Cardiovascular medication includes: regulators of blood pressure and anticoagulants.

Diabetic medication includes: insulin derivatives and DM type 2 variants (e.g. metformin, tolbutamide).

Obstructive lung disease is defined as COPD and/or asthma.

RFA was performed in 47 patients if partial liver resection was not able to render the liver tumor-free. More specifically, RFA was performed because of bilobar metastases (n = 28), risk of insufficient liver function after resection (comorbidity, age or chemotherapy induced liver parenchymal damage, n = 11) or tiny remnant metastases after neoadjuvant chemotherapy for which resection was considered target overshoot (n = 8). The average number of ablated lesions per patient was 1.81 (SD 1.26, range 1–7) and the average size of the largest RFA-treated lesion was 16.4 mm (SD 9.5 mm).

The median follow-up in the patients who underwent simultaneous treatment was 25.5 (IQR = 9.43–49.53) months. At the end of the follow-up period, 47/106 (44.3%) were alive without recurrent disease, 26/106 (24.5%) were alive with recurrent disease and 33/106 (31.1%) patients were deceased. The in-hospital mortality was 3/106 (2.8%), all in the liver resection group. Two out of 47 patients (4.3%) treated by RFA developed an ablation site recurrence after 8 and 23 months, which were re-treated by liver resection or re-ablation, both with curative intent. The five-year overall survival of all 106 patients undergoing simultaneous treatment was 54.3% with a median overall survival of 70.2 months (95%CI = 43.0–97.5). S1 Table shows all complications registered and categorized in liver-related, bowel-related and general complications stratified by type of liver treatment.

### Multivariate analysis of complication rate and severity in simultaneous treatment

In this study, we separately analyzed the complication rate and the complication severity in patients undergoing simultaneous treatment (Tables 2 and 3). To this end we performed a regression analysis to determine the risk factors for developing any complication (complication rate, Table 2). Secondly, we performed a regression analysis to determine the risk factors for developing a high CCI only in the group of patients who developed complications (complication severity, Table 3). In total, 63 of 106 patients (59.4%) suffered from complications. Table 2 shows that patients undergoing an APR (p = 0.027) and patients older than 60 years (p = 0.022) have a higher complication rate. With respect to the treatment of the primary tumor, patients who underwent an APR (20/24) more often suffered complications compared to low anterior resection (LAR) (25/48; p = 0.01) or colon treatment (18/34; p = 0.016). Of note, in univariate analysis of patients that underwent RFA, the diameter of the ablated metastases was larger in patients with complications (18.6mm ± 10.5) versus those without complications (12.8mm ± 6.4, p = 0.023).

**Table 2:** Regression analysis of complication rate.

	Univariable analysis			Multivariable analysis		
Factors	P value	HR	95% CI	P value	HR	95% CI
**Patient characteristics**						
Female sex	0.244	0.628	0.287–1.373			
Age > 60 years	0.068	2.095	0.946–4.639	0.022	3.118	1.176–8.262
BMI > 30	0.638	0.977	0.885–1.078			
Current smoking	0.741	0.848	0.320–2.248			
**Comorbidity**						
ASA score ≥ 3	0.260	2.020	0.595–6.861			
Cardiovascular medication	0.224	1.635	0.741–3.607			
Diabetic medication	0.979	1.025	0.164–6.407			
Syst. corticosteroid medication	0.248	3.621	0.408–32.140			
Obstructive lung disease	0.070	7.000	0.853–57.448	0.057	8.231	0.936–73.999
**Tumor characteristics**						
High CRS (3–5) [21]	0.728	1.148	0.527–2.502			
Bilobar disease	0.615	1.238	0.539–2.845			
**Treatment**						
Neoadjuvant chemo	0.405	0.704	0.308–1.608			
> 1 liver segment surgery	0.146	1.075	0.445–2.598	0.079	2.422	0.902–6.502
Major liver surgery	0.872	1.808	0.814–4.018			
RFA performed	0.979	1.011	0.463–2.206			
APR performed	0.010	4.535	1.425–14.433	0.027	4.382	1.180–16.277
Operation > 8 hours	0.008	2.986	1.333–6.688	0.489	1.417	0.528–3.802
Blood loss > 500ml	0.167	1.768	0.788–3.968	0.315	1.671	0.613–4.554

A binary logistic regression analysis was performed with complication rate as the dependent variable (n = 106). Variables with a p-value < 0.17 were entered in the multivariable analysis.

RFA = radiofrequency ablation, APR = abdominoperineal resection, CRS = clinical risk score, BMI = body mass index, ASA-score = American Society of Anaesthesiologists physical status classification system.

**Table 3:** Regression analysis of the comprehensive complication index (CCI) of patients with complications.

	Univariable analysis	Multivariable analysis	
Factors	P value	P value	Standardized Beta
**Patient characteristics**			
Female sex	0.618		
Age > 60 years	0.762		
BMI > 30	0.239		
Current smoking	0.383		
**Comorbidity**			
ASA score ≥ 3	0.259		
Cardiovascular medication	0.874		
Diabetic medication	0.712		
Syst. corticosteroid medication	0.138	0.290	-0.134
Obstructive lung disease	0.323		
**Tumor characteristics**			
High CRS (3–5)[21]	0.233		
Bilobar disease	0.498		
**Treatment**			
Neoadjuvant chemo	0.458		
> 1 liver segment surgery	0.993		
Major liver surgery	0.462		
RFA performed	0.021	0.040	-0.263
APR performed	0.306		
Operation > 8 hours	0.377		
Blood loss > 500ml	0.421		

A linear regression was performed in all patients who developed complications (n = 63), with CCI score as the dependent variable. Variables with a p-value < 0.17 were entered in the multivariable analysis.

RFA = radiofrequency ablation, APR = abdominoperineal resection, CRS = clinical risk score, BMI = body mass index, ASA-score = American Society of Anaesthesiologists physical status classification system.

When comparing the CCI in the patients who actually suffered from complications, univariable analysis showed that RFA-treated patients had a lower complication severity (27.9 ± 13.0) compared to non-RFA-treated patients. (39.6 ± 23.3; p = 0.021). This difference in complication severity maintained significance in the multivariable analysis (p = 0.040; Table 3).

### Survival in matched analysis simultaneous vs. colorectal-first

**Fig 1:**
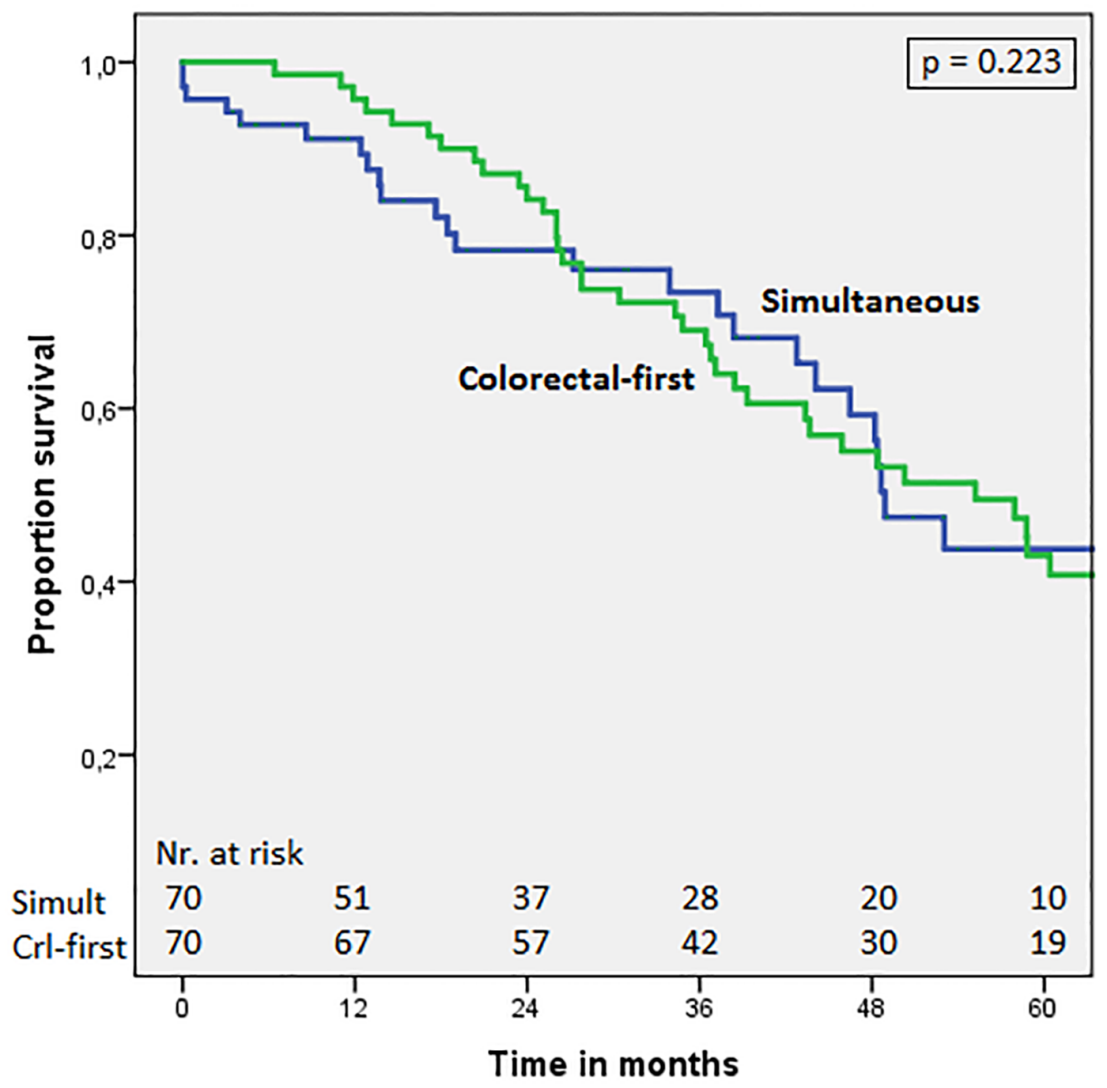
Overall survival in matched patients: Simultaneous treatment vs. colorectal-first. Comparison of patients with synchronous CRLM undergoing the simultaneous treatment or the colorectal-first approach. P-value = 0.223 (stratified log-rank test).

To confirm the findings of previous research of comparable survival in the simultaneous versus the colorectal-first approach, we performed a matched analysis. A matched pair analysis was performed to reduce bias due to confounding variables. Seventy patients who underwent colorectal-first surgery were successfully matched to 70 patients (70/106; 66.0%) who underwent simultaneous treatment. Table 4 shows the clinicopathological characteristics of matched patients with synchronous liver metastases undergoing simultaneous versus colorectal-first treatment. Fig 1 shows comparable survival curves in the simultaneous group and colorectal-first group, with 5-year overall survival of 43.8% and 43.0% and median overall survival of 48.9 months (95%CI = 42.8–55.0) and 55.2 months (95%CI = 41.7–68.7), respectively (p = 0.223).

**Table 4:** Clinicopathological characteristics of matched patients with synchronous liver metastases undergoing simultaneous or colorectal-first treatment.

	Simultaneous (n = 70)	Colorectal first (n = 70)	P-value
**Patient characteristics**			
Mean age ± SD	62.2 ± 11.6	62.3 ± 9.0	0.947
Male gender	37 (52.9%)	34 (48.6%)	0.720
**Extent of liver surgery**			0.475
≥3 segments	25 (35.7%)	27 (38.6%)	
1 or 2 segments	13 (18.6%)	14 (20.0%)	
RFA or wedge resection	32 (45.7%)	29 (41.4%)	
**RFA**			0.424^a^
RFA as part of treatment	30 (42.9%)	25 (35.7%)	
• RFA + resection	19 (29.9%)	11 (15.7%)	
• Only RFA	11 (15.7%)	20.0%)	
Of which percutaneous	0	10 (14.3%)	
**Characteristics tumor**			
Low clinical risk score (0–2)[21]	37 (52.9%)	36 (51.4%)	1.000
Diam. CRLM in cm (median ± IQR)	2.5 ± 2.5	3.0 ± 3.5	0.106
Neoadjuvant chemotherapy	35 (50.0%)	32 (45.7%)	0.690
Primary tumor at rectal site	36 (51.4%)	34 (48.6%)	0.832
Bilobar liver disease	23 (32.9%)	32 (45.7%)	0.160

Matching was performed based on the characteristics: location of primary tumor, major/minor liver surgery, type of liver procedure, clinical risk score, sex, age and neoadjuvant chemotherapy.

P values were calculated using the paired T test, McNemar test or Wilcoxon signed rank test.

^a^ RFA vs. no RFA in simultaneous vs. colorectal-first treatment.

### Survival in matched analysis in RFA with or without liver resection vs. liver resection alone

**Fig 2:**
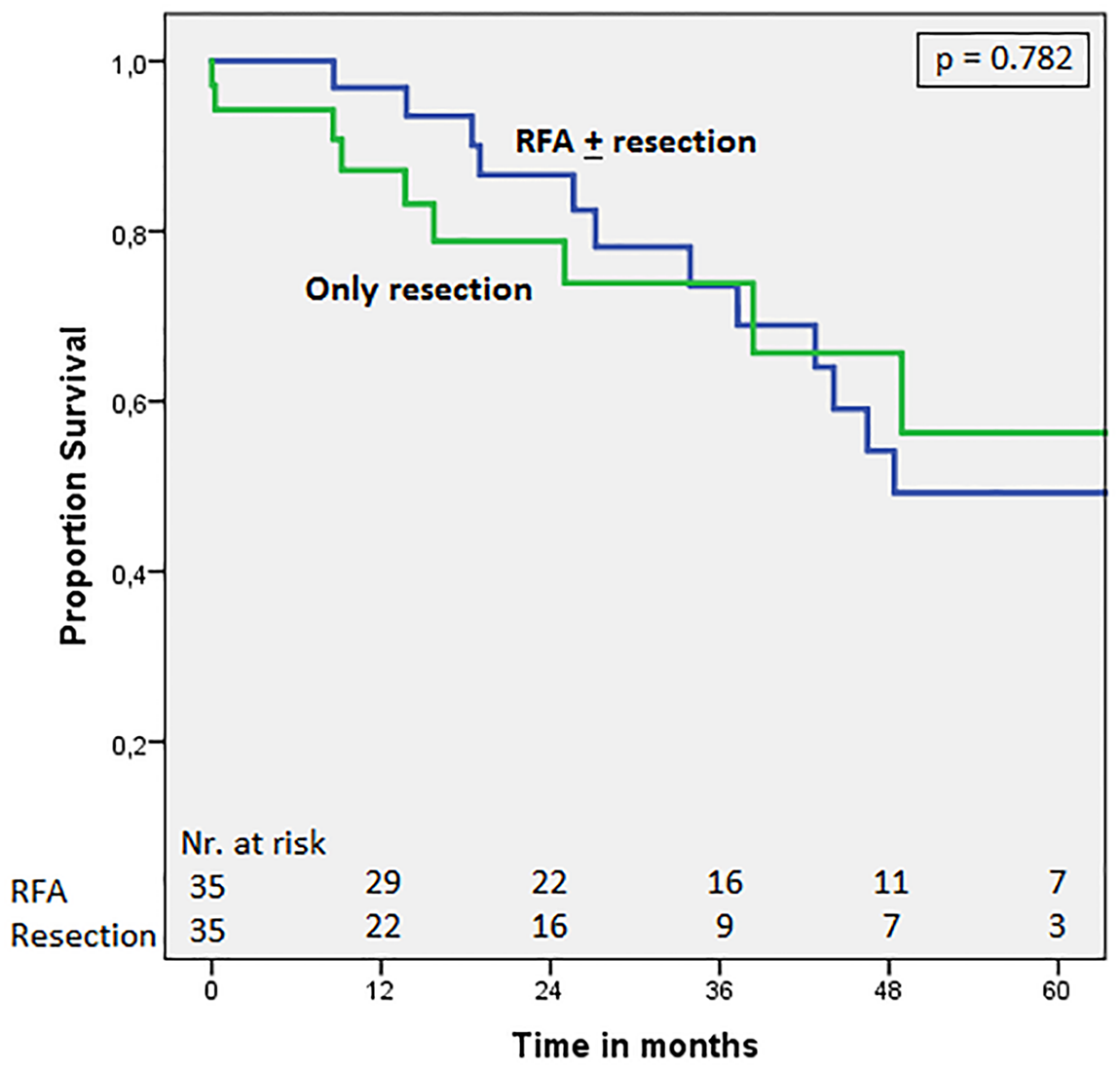
Overall survival in matched patients: RFA ± resection vs. only resection. Comparison of patients with synchronous CRLM undergoing surgical treatment including RFA (RFA ± resection) or only liver resection. P-value = 0.782 (stratified log-rank test).

**Fig 3:**
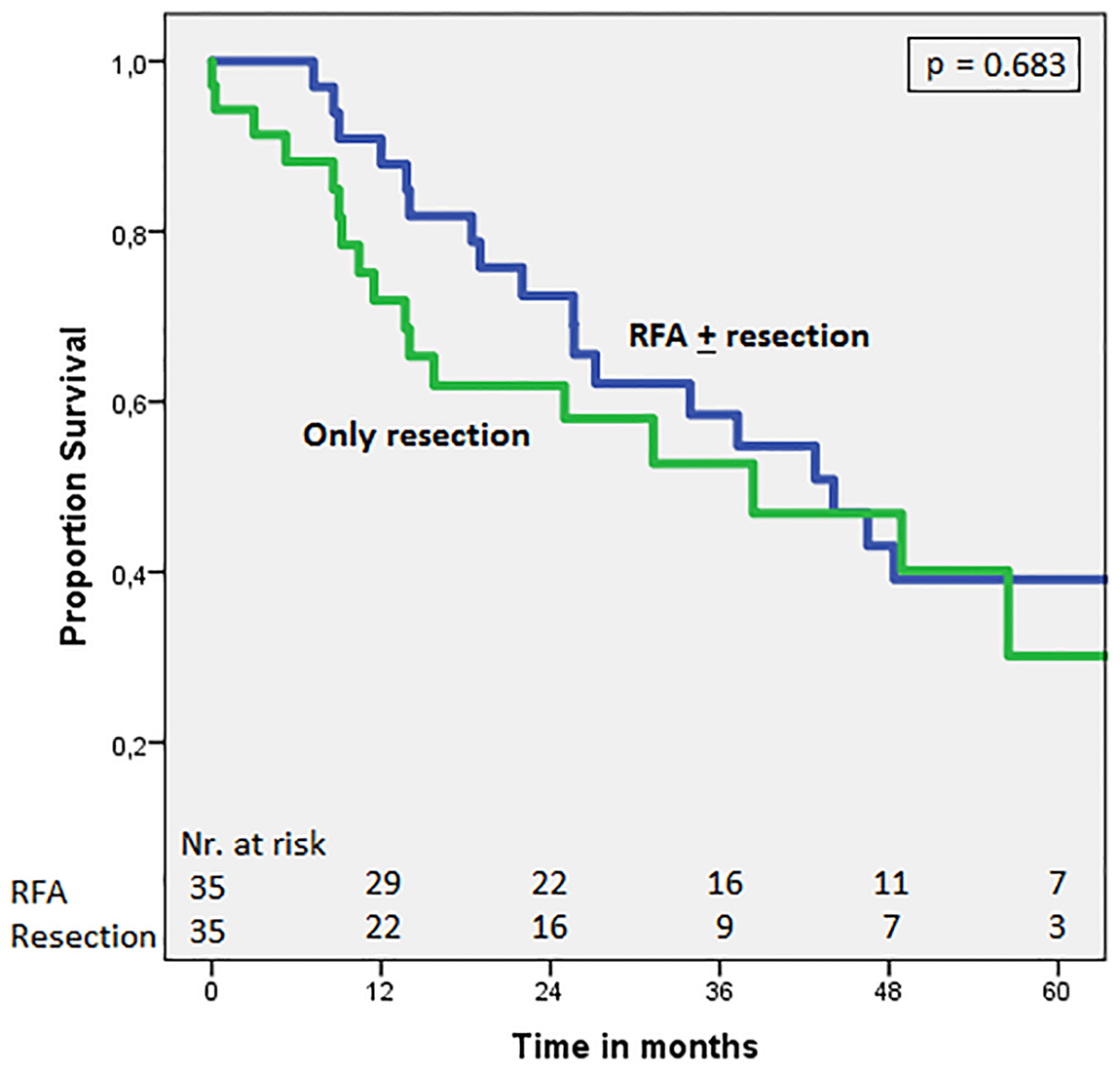
Disease-free survival in matched patients: RFA ± resection vs. only resection. Comparison of patients with synchronous CRLM undergoing surgical treatment including RFA (RFA ± resection) or only liver resection. P-value = 0.683 (stratified log-rank test).

Thirty-five patients (35/47; 74.5%) who underwent a treatment including RFA, either as a sole treatment or in combination with liver resection (RFA ± liver resection treatment), were successfully matched to 35 patients in whom no RFA was applied and only underwent liver resection. Table 5 shows the clinicopathological characteristics of all matched patients. Fig 2 shows comparable survival curves in the RFA group and the liver resection group, with 5 year overall survival of 49.2% and 56.3% and median overall survival of 48.4 months (95%CI = 18.3–78.4) and 70.2 months (95%CI = 31.1–109.3), respectively (p = 0.782). Likewise, Fig 3 shows comparable survival curves in the RFA group and the liver resection group concerning disease-free survival (DFS), with a 5 year DFS of 39.1% and 30.1% and a median DFS of 44.1 months (95%CI = 29.2–59.0) and 38.4 months (95%CI = 11.7–65.1), respectively (p = 0.683).

**Table 5:** Clinicopathological characteristics of matched patients undergoing RFA as a part of treatment vs. liver resection only.

	Liver resection (n = 35)	RFA ± resection (n = 35)	P-value
**Patient characteristics**			
Mean age ± SD	59.6 ± 12.2	64.2 ± 10.6	0.037
Male sex	20 (57.1%)	20 (57.1%)	1.000
**Extent of liver intervention**			0.549 ^a^
≥3 segments	6 (17.1%)	5 (14.3%)	
1 or 2 segments	6 (17.1%)	6 (17.1%)	
Wedge resection	23 (65.7%)	15 (42.8%)	
Only RFA	-	9 (25.7%)	
**Type of colorectal surgery**			0.228
Abdominoperineal resection	8 (22.9%)	8 (22.9%)	
Low anterior resection	14 (40.0%)	16 (45.7%)	
Colon	13 (37.1%)	11 (31.4%)	
**Characteristics tumor**			
Low clinical risk score (0–2) [21]	20 (57.1%)	22 (62.9%)	0.774
Diam. CRLM in cm (median ± IQR)	2.2 ± 2.0	1.9 ± 1.8	0.268
Neoadjuvant chemotherapy	24 (68.6%)	25 (71.4%)	1.000
Primary tumor at rectal site	23 (65.7%)	24 (68.6%)	1.000
Bilobar disease	6 (37.1%)	23 (65.7%)	<0.001

Matching was performed based on the characteristics: type of colorectal surgery, major/minor liver surgery, clinical risk score, age and neoadjuvant chemotherapy.

^a^ McNemar test for a 3x3 comparison of ‘>3 segments’ vs. ‘1 or 2 segments’ vs. ‘wedge/RFA’.

P values were calculated using the paired T test, McNemar test or Wilcoxon signed rank test.

## Discussion

The main finding of this study in patients with synchronous colorectal liver metastases who underwent simultaneous surgery of the primary tumor and its liver metastases, is that the CCI, as a comprehensive score of all occurring complications including their weighted severity, showed that RFA had a lower complication severity compared to liver resection only. Additionally, RFA treatment of the liver metastases—either alone or as an adjunct to liver resection- has a comparable overall survival and disease-free survival as liver resection alone. Of note, the surgical procedure performed for treatment of the primary tumor (APR) and a patient characteristic (age > 60) were associated with a higher complication rate. To corroborate findings as published by most other medical centers, we could also demonstrate in a matched analysis that patients undergoing simultaneous surgery had a similar overall survival compared to patients undergoing colorectal-first surgery. Of note, two out of nine centers in the review by Siriwardena et al. presented a worse survival for the patients undergoing simultaneous surgery compared to staged resection [24].

There is considerable variation in survival rates among studies comparing RFA with liver resection, both for the overall and the disease free survival [13,15,25–27]. In our study, almost half of patients undergoing simultaneous treatment were treated with RFA. Twenty-eight out of the 47 RFA-treated patients underwent RFA because of bilobar liver metastases. The other treatment option for bilobar CRLM is a two-stage hepatectomy, in which around 30% of the patients planned for a 2-stage hepatectomy actually never undergo the second procedure [28–30]. In a recent study with matched patients -matching based on oncological prognostic markers-, survival rate in a two-stage hepatectomy was compared to a one-stage hepatectomy [31]. In the latter patient group, parenchyma-sparing, ultrasound guided liver surgery was performed with complete tumor clearance in one procedure. The authors found that drop out (38.1%) in the two-stage group was not caused by selection of patients with oncological more aggressive tumors, but by the inability to obtain complete liver tumor clearance in one procedure. In parallel, we suggest that the use of RFA to obtain complete tumor clearance in one procedure in these patients will have the best chance on intentional curative treatment.

Progression of the CRLM has been observed after removal of the primary tumor, which suggests that simultaneous resection of both the colorectal primary and the liver tumor might yield better oncological outcome [32–34]. Two large multicentre studies [1,6] and our results show that the overall survival comparing colorectal-first and simultaneous strategies is similar. A recent study analyzed the effect of resection of the primary tumor on synchronous liver metastases with a multivariate model to predict progressive disease, in which the only adverse prognostic variable was undergoing an upfront primary colorectal resection [35].

In our study, the extent of liver surgery in simultaneous procedures did not substantially contribute to the rate and severity of complications. This favorable complication pattern may be due to selection of patients, since the combination of APR with major liver resection was scarce. Our results of a lower complication rate in RFA patients with simultaneous treatment is in concordance with a consensus statement on RFA treatment in general as compared to liver resection [12]. Concerning treatment of the primary colorectal cancer, we and others show both higher complication rates in patients undergoing APR, compared to both LAR and colon surgery [36–38].

A limitation of this study is its retrospective design. This study, however has its merits because it describes an observation of clinical decision-making. We realize that this study has limited power due to the number of patients, however, this is the largest study to date analyzing RFA in simultaneous resections.

In conclusion, patients who underwent RFA of liver metastases show similar oncological outcome and lower complication severity compared to liver resection in simultaneous treatment of both the primary colorectal carcinoma and the liver metastases. Hence, RFA is a useful treatment option in simultaneous resections.

## Supporting information

S1 Table All complications registered. All complications registered, stratified by both treatment (liver resection, resection + RFA and RFA) and by (anatomical) reason of complications (bowel-related, liver-related, general).
(DOCX)
