# Supplementary material for: Radiofrequency ablation is beneficial in simultaneous treatment of synchronous liver metastases and primary colorectal cancer
Source: PLoS One. 2018 Mar 15;13(3):e0193385. doi: 10.1371/journal.pone.0193385 (PMC5854258; doi:10.1371/journal.pone.0193385)
Supplement: S1 Table — All complications registered, stratified by both treatment (liver resection, resection + RFA and RFA) and by (anatomical) reason of complications (bowel-related, liver-related, general). (DOCX) [file pone.0193385.s001.docx]

|  | **Bowel complications** | **X** | **Liver complications** | **X** | **General complications** | **X** |
| --- | --- | --- | --- | --- | --- | --- |
| **Liver resection**  **(n = 59)** | anastomotic leakage/ abscess | 6 | biloma | 3 | electrolytes disbalance | 5 |
|  | ileus | 5 | portal vein thrombosis | 1 | urinary tract infection | 4 |
|  | high-output enterostoma | 2 | liver abscess | 1 | spf. wound infection | 4 |
|  | coprostasis | 1 | bile leakage | 1 | atrial fibrillation | 3 |
|  | intra-abdominal bleeding | 1 |  |  | delirium | 3 |
|  | intra-abdominal abscess | 1 |  |  | gastroparesis | 3 |
|  | death (intra-abdominal abscess) | 1 |  |  | infected ascites | 3 |
|  |  |  |  |  | anemia | 3 |
|  |  |  |  |  | chylous effusion | 3 |
|  |  |  |  |  | renal failure | 2 |
|  |  |  |  |  | cardiac failure | 2 |
|  |  |  |  |  | platzbauch | 2 |
|  |  |  |  |  | pneumonia | 2 |
|  |  |  |  |  | brachial plexus injury | 1 |
|  |  |  |  |  | urinary retention | 1 |
|  |  |  |  |  | death (respiratory) | 1 |
|  |  |  |  |  | acute pleural effusion | 1 |
|  |  |  |  |  | oral candidiasis | 1 |
|  |  |  |  |  | pulmonary embolism | 1 |
|  |  |  |  |  | death (hemodynamic instability) | 1 |
|  |  |  |  |  | fluid overload | 1 |
|  |  |  |  |  |  |  |
| **Resection+ RFA**  **(n = 34)** | anastomotic leakage/ abscess | 3 | infected RFA ablation zone | 1 | spf. wound infection | 4 |
|  | intra-abdominal bleeding | 2 | portal vein thrombosis | 1 | delirium | 2 |
|  | intra-abdominal abscess | 1 | intrahepatic cyst | 1 | gastroparesis | 2 |
|  | death (respiratory/abdominal)* | 1 | liver abscess | 1 | urinary retention | 2 |
|  |  |  |  |  | infected seroma | 1 |
|  |  |  |  |  | urinary tract infection | 1 |
|  |  |  |  |  | platzbauch | 1 |
|  |  |  |  |  | atrial fibrillation | 1 |
|  |  |  |  |  | hypertension | 1 |
|  |  |  |  |  | pneumonia | 1 |
|  |  |  |  |  | death (respiratory/abdominal)* | 1 |
|  |  |  |  |  | acute pleural effusion | 1 |
|  |  |  |  |  |  |  |
| **RFA**  **(n = 13)** | anastomotic leakage/ abscess | 2 |  |  | urinary retention | 2 |
|  | ileus | 1 |  |  | pneumonia | 2 |
|  | peri-anal wound infection | 1 |  |  | spf. wound infection | 2 |
|  | high-output enterostoma | 1 |  |  | SIRS | 1 |
|  |  |  |  |  | hypertension | 1 |
